# Supplementary material for: Estimating mosquito bionomics parameters with a hierarchical Bayesian model
Source: Curr Res Parasitol Vector Borne Dis. 2026 Jul 11;10:100411. doi: 10.1016/j.crpvbd.2026.100411 (PMC13383950; doi:10.1016/j.crpvbd.2026.100411)
Supplement: Supplementary file 1 — Supplementary Tables S1-S2 and Supplementary Figures S1-S21. [file mmc1.pdf]

Estimating mosquito bionomics parameters with a hierarchical Bayesian model

Supplementary file 1

Jeanne Lemant[1,2*], Aurélien Tarroux[1,2*], Thomas A Smith[1,2], Barnabas Zogo[3], Monica Golumbeanu[1,2], Olukayode G. Odufuwa[1,2,4], Seth Irish[5], Sarah Moore[1,2,4], Emilie Pothin[1,2,6], Clara Champagne[1,2]

[1] Swiss Tropical and Public Health Institute, Allschwil, Switzerland
[2] University of Basel, Basel, Switzerland
[3] University of Montpellier, Montpellier, France
[4] Ifakara Health Institute, Bagamoyo, Tanzania
[5] World Health Organization, Geneva, Switzerland
[6] Clinton Health Access Initiative, Boston, United States of America
* Equal contribution

Contents

1. Taxonomy 2

2. Bionomic parameter estimation 4

Endophagy 4

Endophily 8

Human Blood Index 9

Resting Duration 14

Sac rate 16

3. Comparison of Bayesian estimates with empirical means 19

4. Vectorial Capacity reduction 20

5. References 22

1. Taxonomy

Category name	Species
Albitarsis complex	*Anopheles albitarsis A*<br>*Anopheles albitarsis B*<br>*Anopheles albitarsis E*<br>*Anopheles marajoara*
Annularis complex	*Anopheles annularis A*<br>*Anopheles philippinensis*
Barbirostris complex	*Anopheles barbirostris*
Dirus complex	*Anopheles baimaii*<br>*Anopheles cracens*<br>*Anopheles dirus*
Funestus group	*Anopheles aconitus*<br>*Anopheles culicifacies A*<br>*Anopheles culicifacies B*<br>*Anopheles culicifacies C*<br>*Anopheles culicifacies D*<br>*Anopheles culicifacies E*<br>*Anopheles flavirostris*<br>*Anopheles fluviatilis*<br>*Anopheles fluviatilis S*<br>*Anopheles fluviatilis T*<br>*Anopheles funestus*<br>*Anopheles harrisoni*<br>*Anopheles minimus*
Gambiae complex	*Anopheles arabiensis*<br>*Anopheles gambiae s.s. / An. coluzzii*<br>*Anopheles melas*<br>*Anopheles merus*
Hyrcanus group	*Anopheles anthropophagus*<br>*Anopheles sinensis*
Jamesii group	*Anopheles jamesii*<br>*Anopheles pseudojamesii*<br>*Anopheles splendidus*
Leucosphyrus complex	*Anopheles balabacensis*<br>*Anopheles latens*<br>*Anopheles leucosphyrus*
Maculatus group	*Anopheles maculatus*
Nili complex	*Anopheles carnevalei*<br>*Anopheles nili*<br>*Anopheles ovengensis*

Nuneztovari complex	*Anopheles nuneztovari B/C*
Punctulatus group	*Anopheles farauti*<br>*Anopheles koliensis*<br>*Anopheles punctulatus*
Quadrimaculatus subgroup	*Anopheles quadrimaculatus*
Subpictus complex	*Anopheles subpictus B*
Sundaicus complex	*Anopheles epiroticus*<br>*Anopheles sundaicus*
Stand-alone names	*Anopheles albimanus*<br>*Anopheles aquasalis*<br>*Anopheles (Nyssorhynchus) darlingi*<br>*Anopheles freeborni*<br>*Anopheles kochi*<br>*Anopheles moucheti*<br>*Anopheles nivipes*<br>*Anopheles stephensi*<br>*Anopheles tessellatus*<br>*Anopheles vagus*<br>*Pseudopunctipennis* complex

Supplementary Table S1 : Categories for hierarchical model and standalone names

2. Bionomic parameter estimation

For each parameter, when applicable, we first provide a flow chart of the selected studies, illustrating the inclusion and exclusion criteria applied. We then present figures summarizing the available data across complexes and species; in these figures, each dot corresponds to a survey, with the mean value indicated on the x-axis and the number of observations indicated on the y-axis. Finally, we report the posterior densities associated with all species and complexes for which data were available, except for endophily, for which posterior distributions are already shown in the main text.

Endophagy

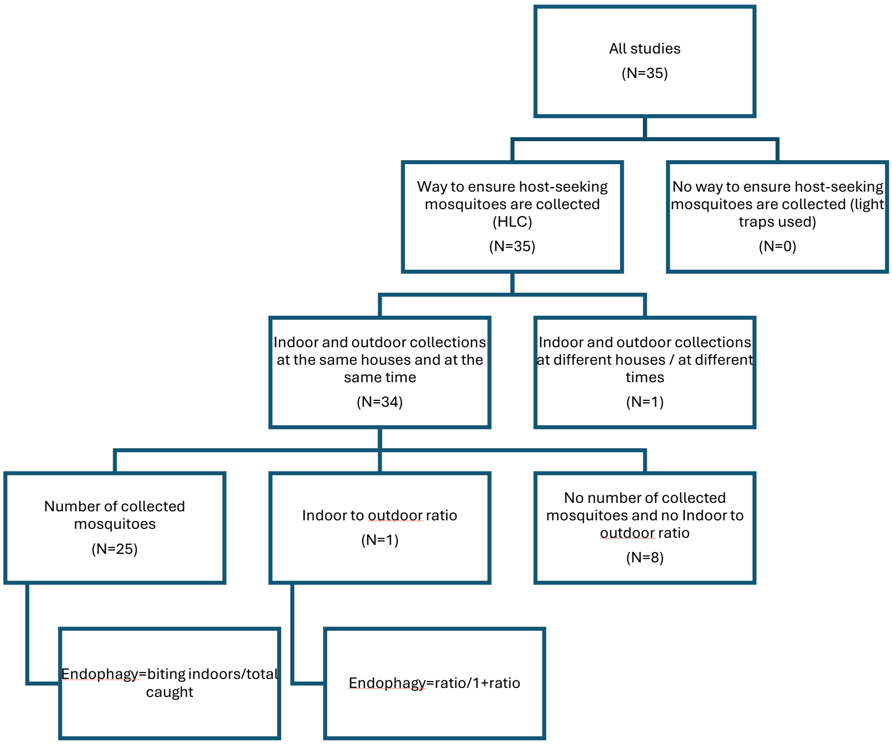

Supplementary Figure S1. Flow chart of selected studies for endophagy, illustrating the inclusion and exclusion criteria applied.

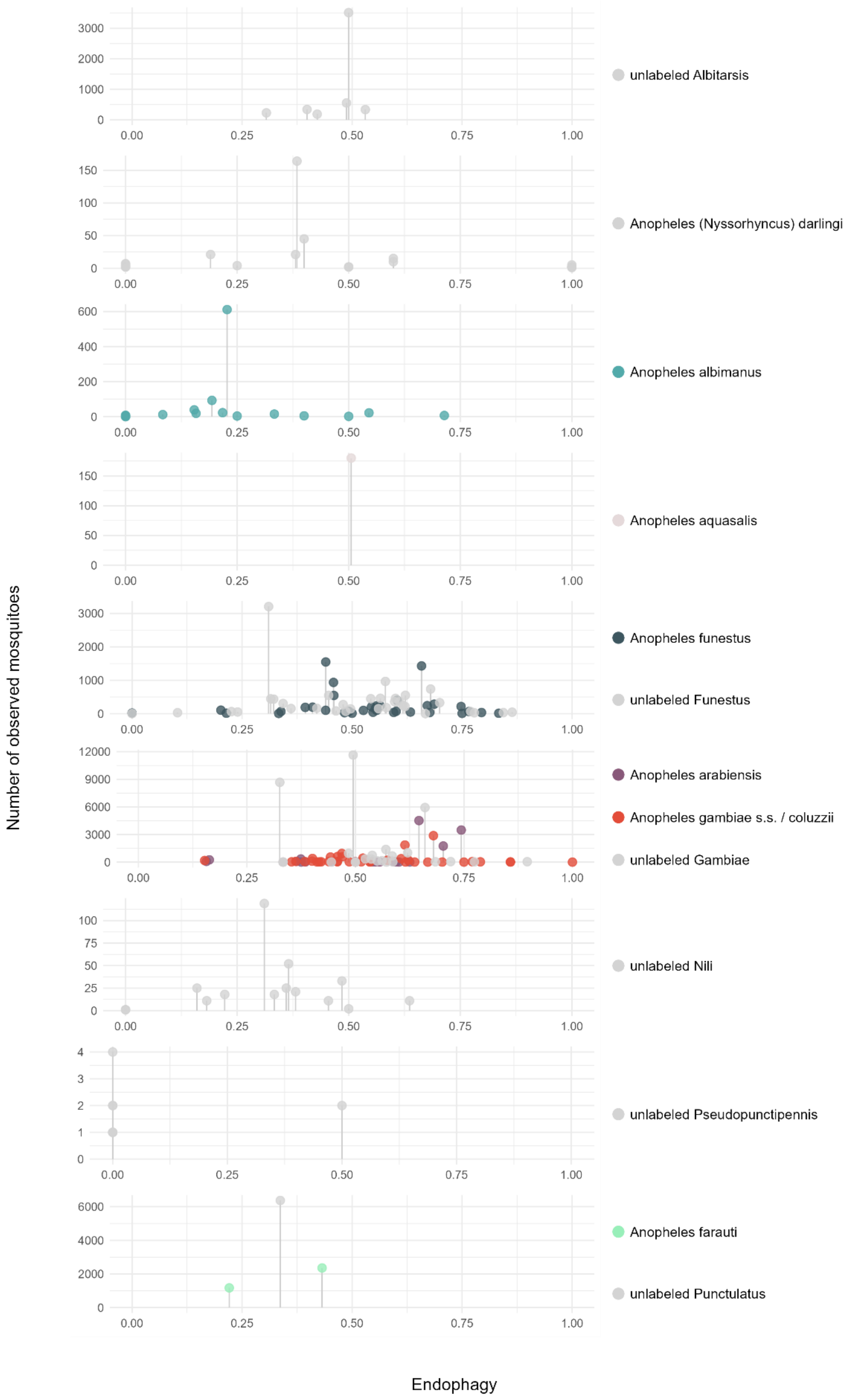

Supplementary Figure S2. Available data by complexes and species for endophagy.

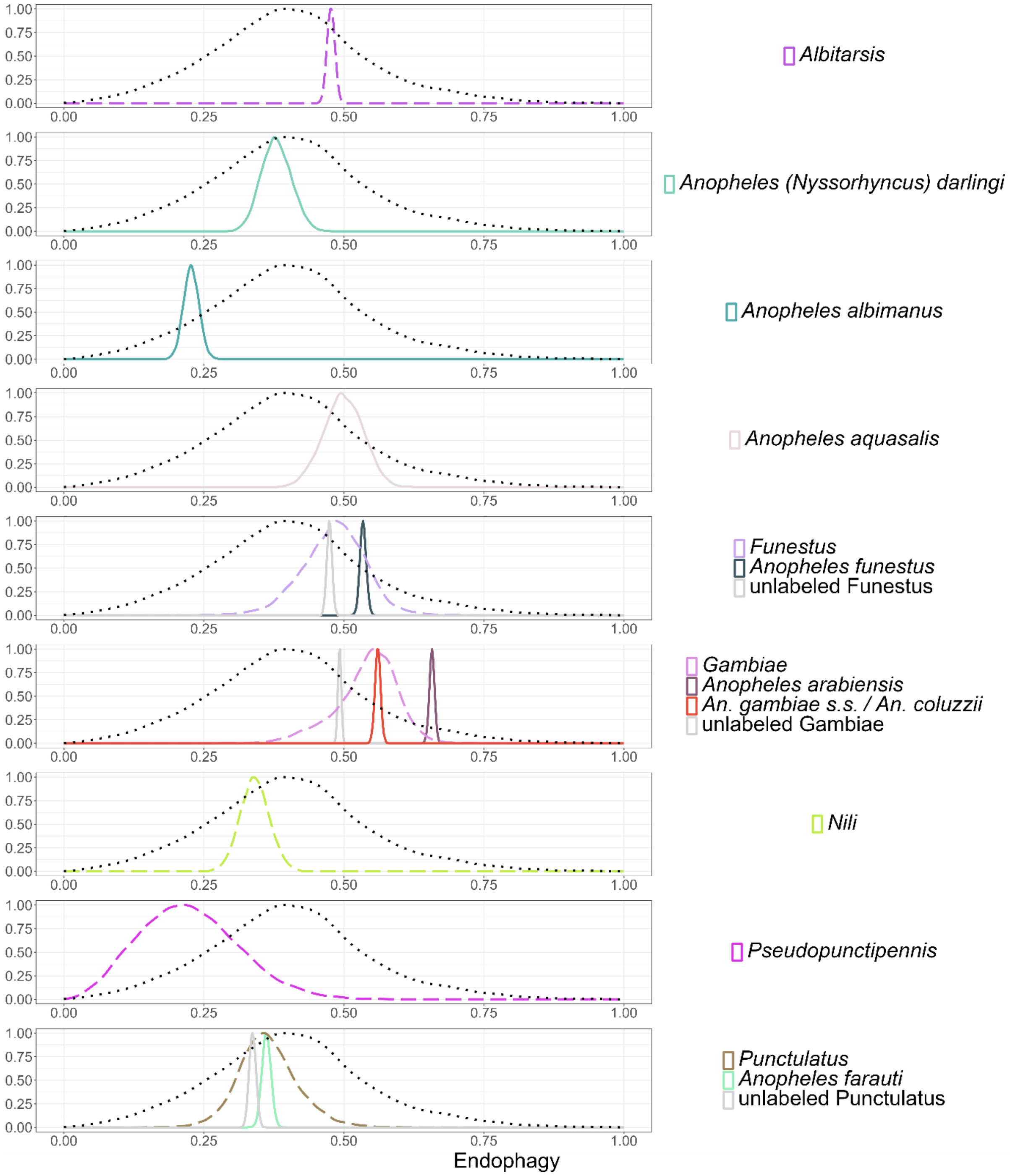

Supplementary Figure S3. Posterior densities for endophagy. The black dotted line is the posterior density for the pooled estimate, the coloured dashed lines, for each complex, and the solid coloured lines for individual species. Y-axis is normalised to ease visualisation of all curves.

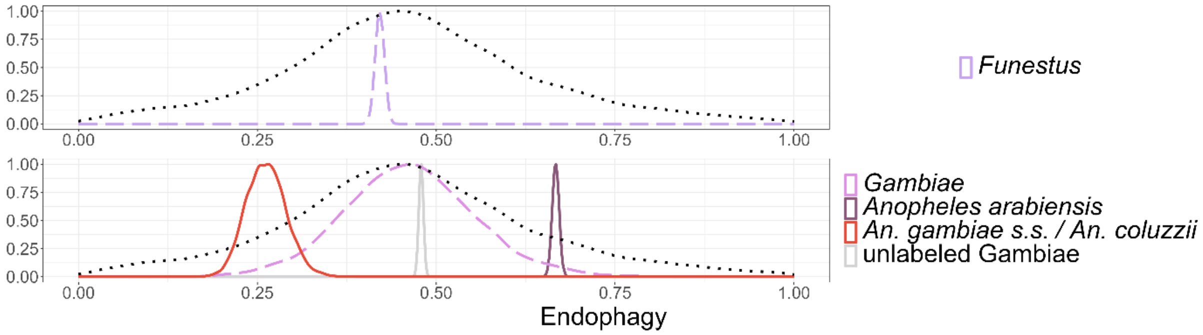

Supplementary Figure S4. Posterior densities for endophagy in East Africa. The black dotted line is the posterior density for the pooled estimate, the coloured dashed lines, for each complex, and the solid coloured lines for individual species. Y-axis is normalised to ease visualisation of all curves.

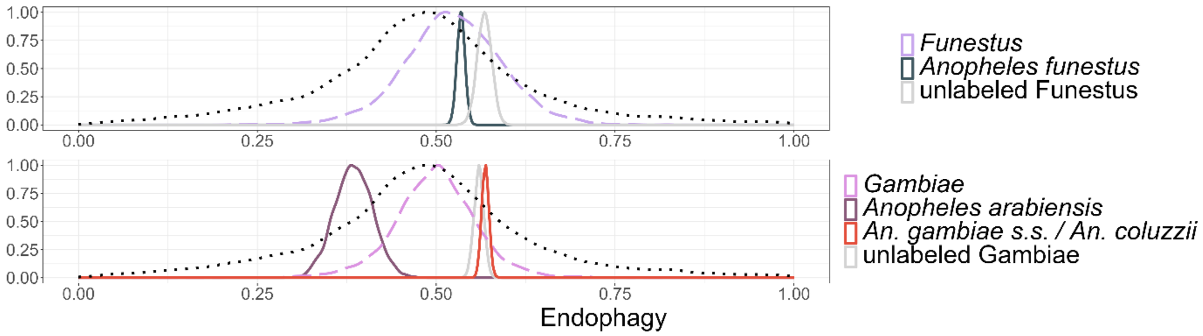

Supplementary Figure S5. Posterior densities for endophagy in West Africa. The black dotted line is the posterior density for the pooled estimate, the coloured dashed lines, for each complex, and the solid coloured lines for individual species. Y-axis is normalised to ease visualisation of all curves.

Endophily

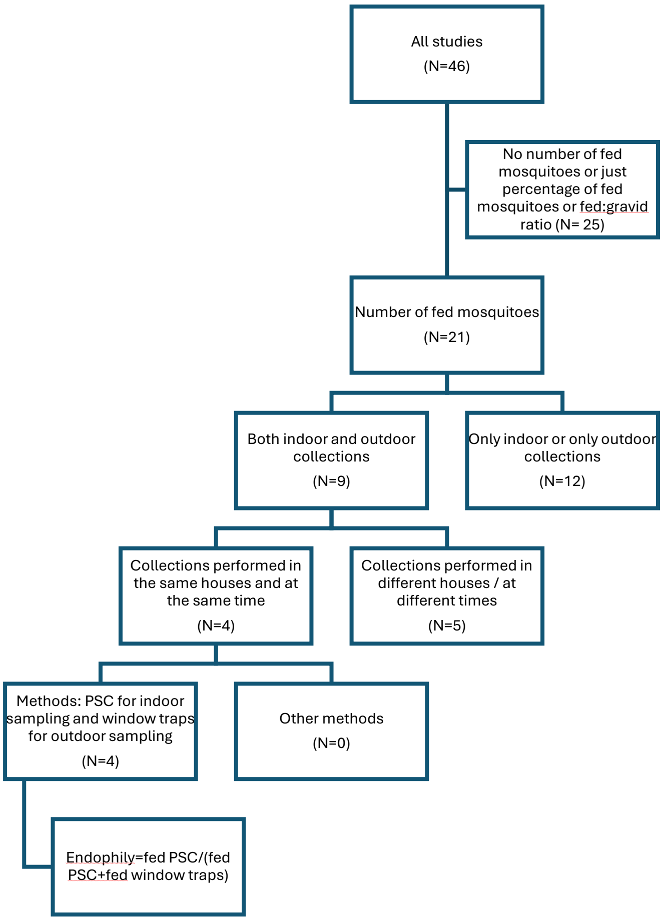

Supplementary Figure S6. Flow chart of selected studies for endophily, illustrating the inclusion and exclusion criteria applied.

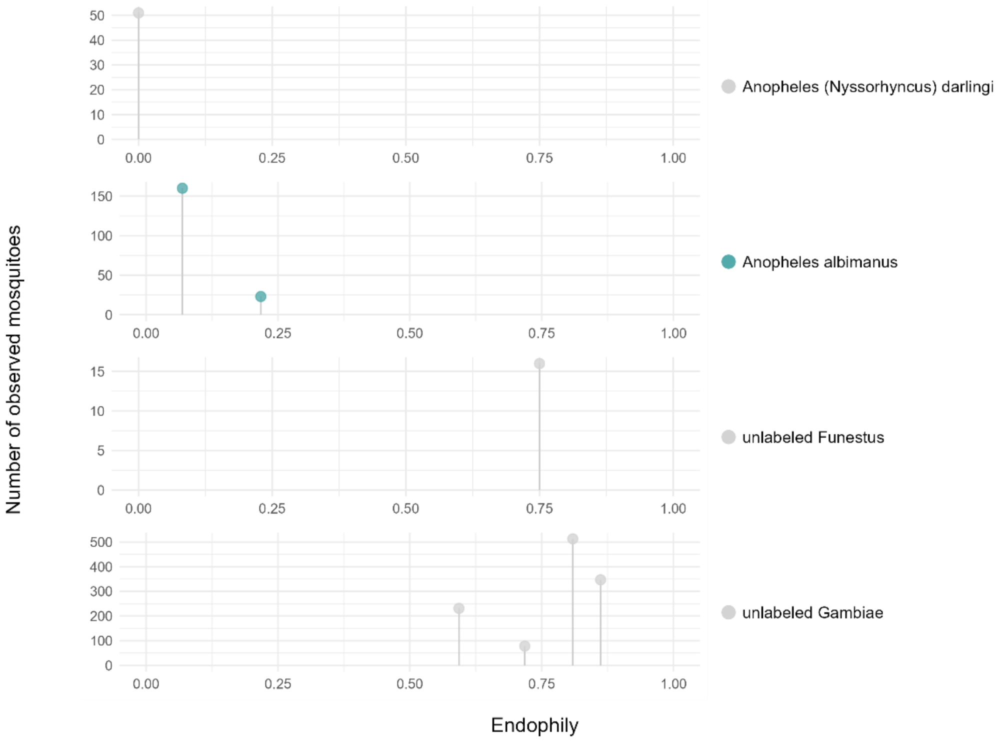

Supplementary Figure S7. Available data by complexes and species for endophily.

Human Blood Index

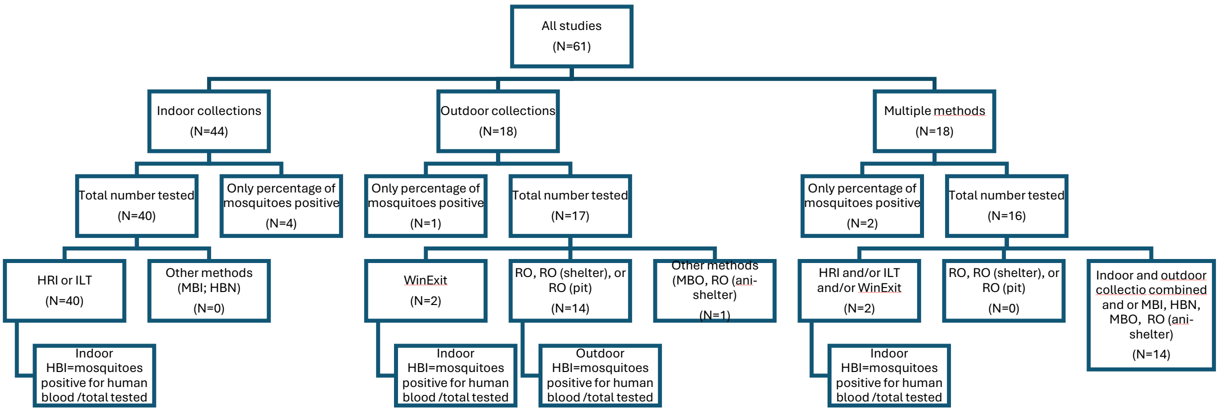

Supplementary Figure S8. Flow chart of selected studies for HBI, illustrating the inclusion and exclusion criteria applied.

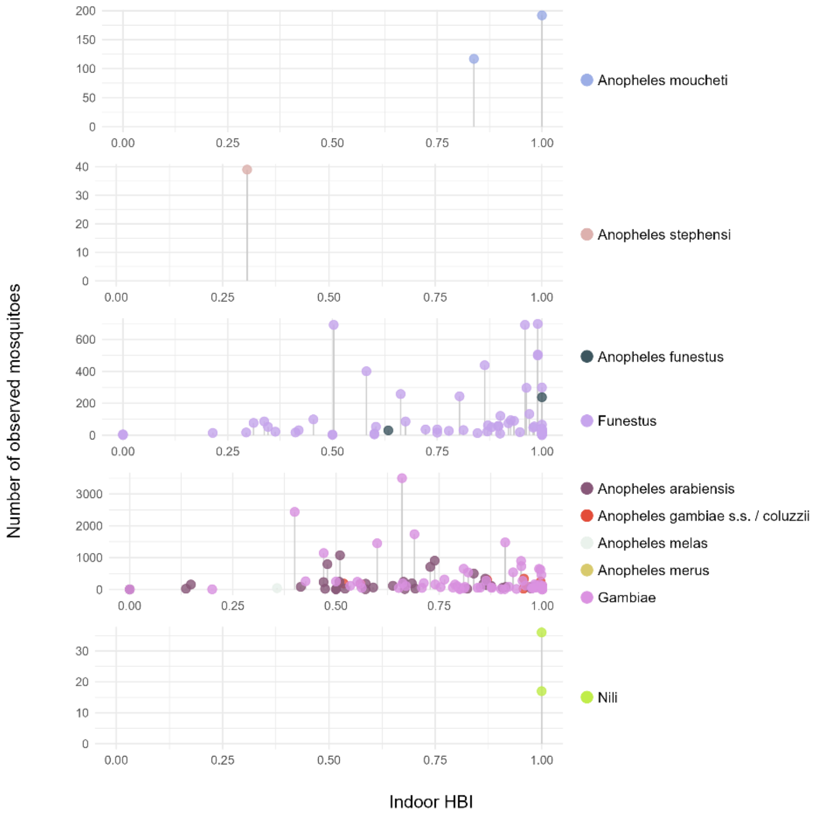

Supplementary Figure S9. Available data by complexes and species for indoor HBI.

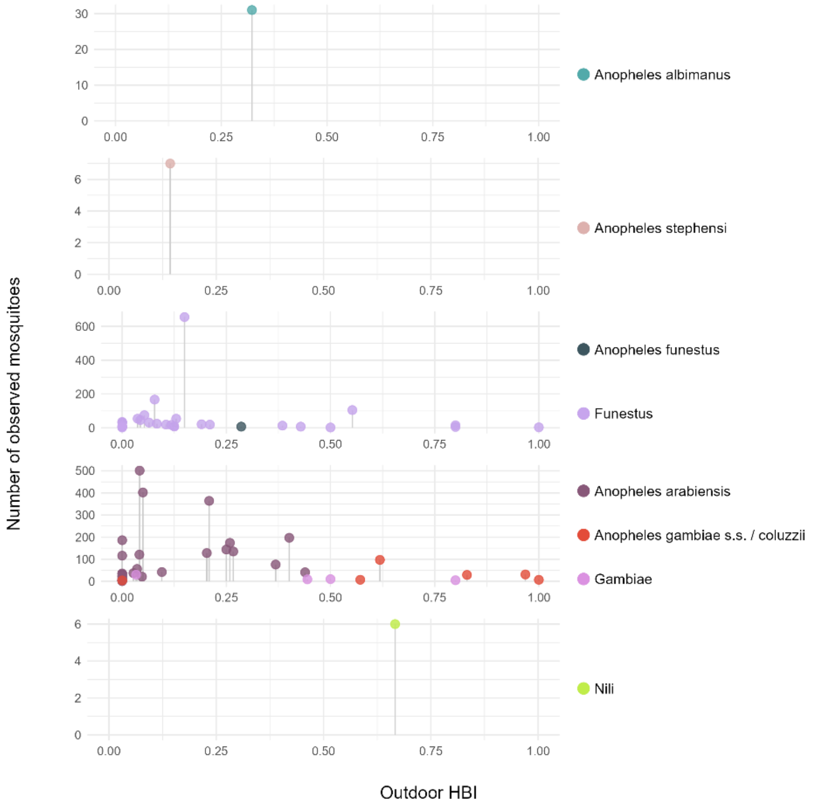

Supplementary Figure S10. Available data by complexes and species for outdoor HBI

Parous rate

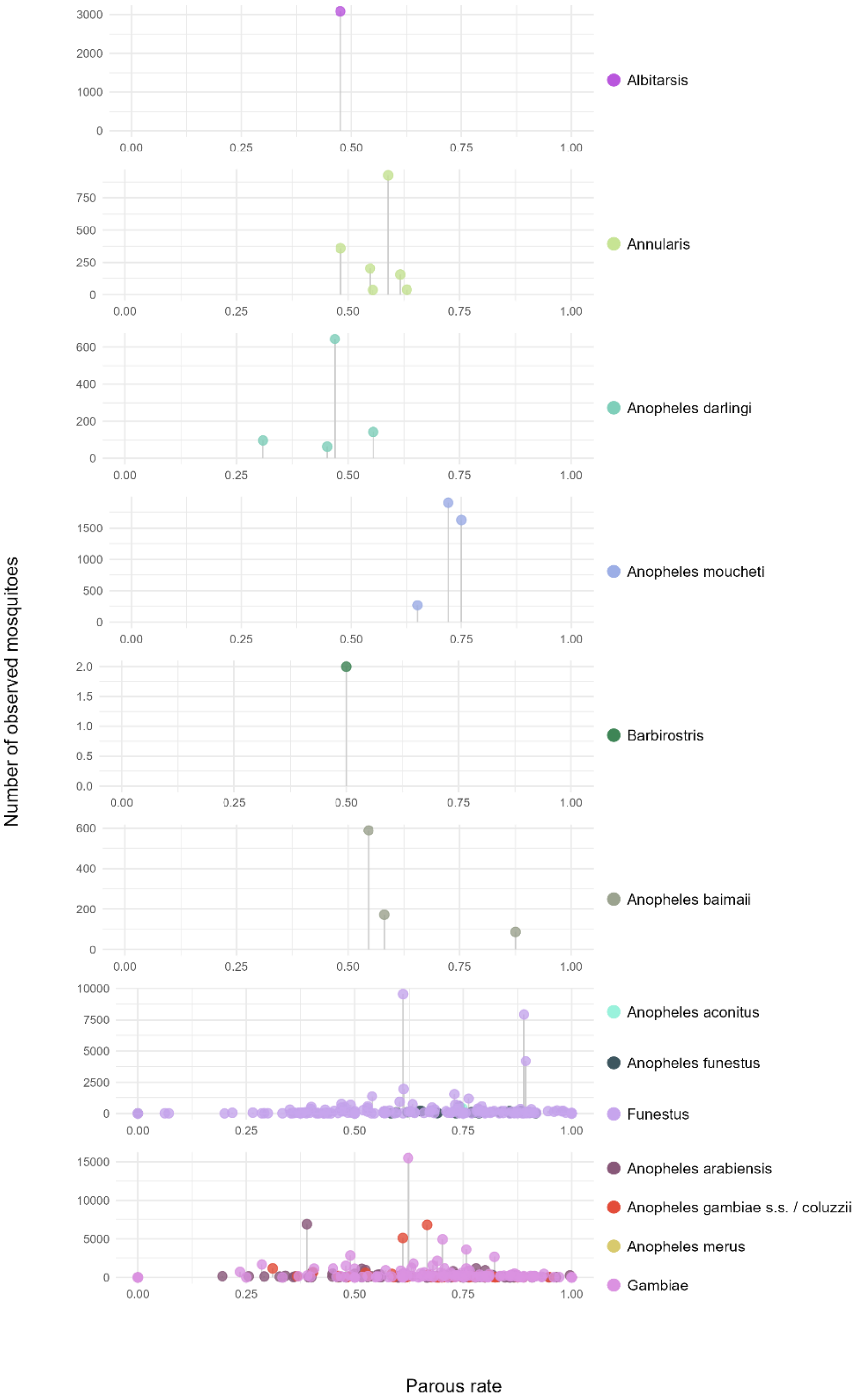

Supplementary Figure S11. Available data by complexes and species for parous rate.

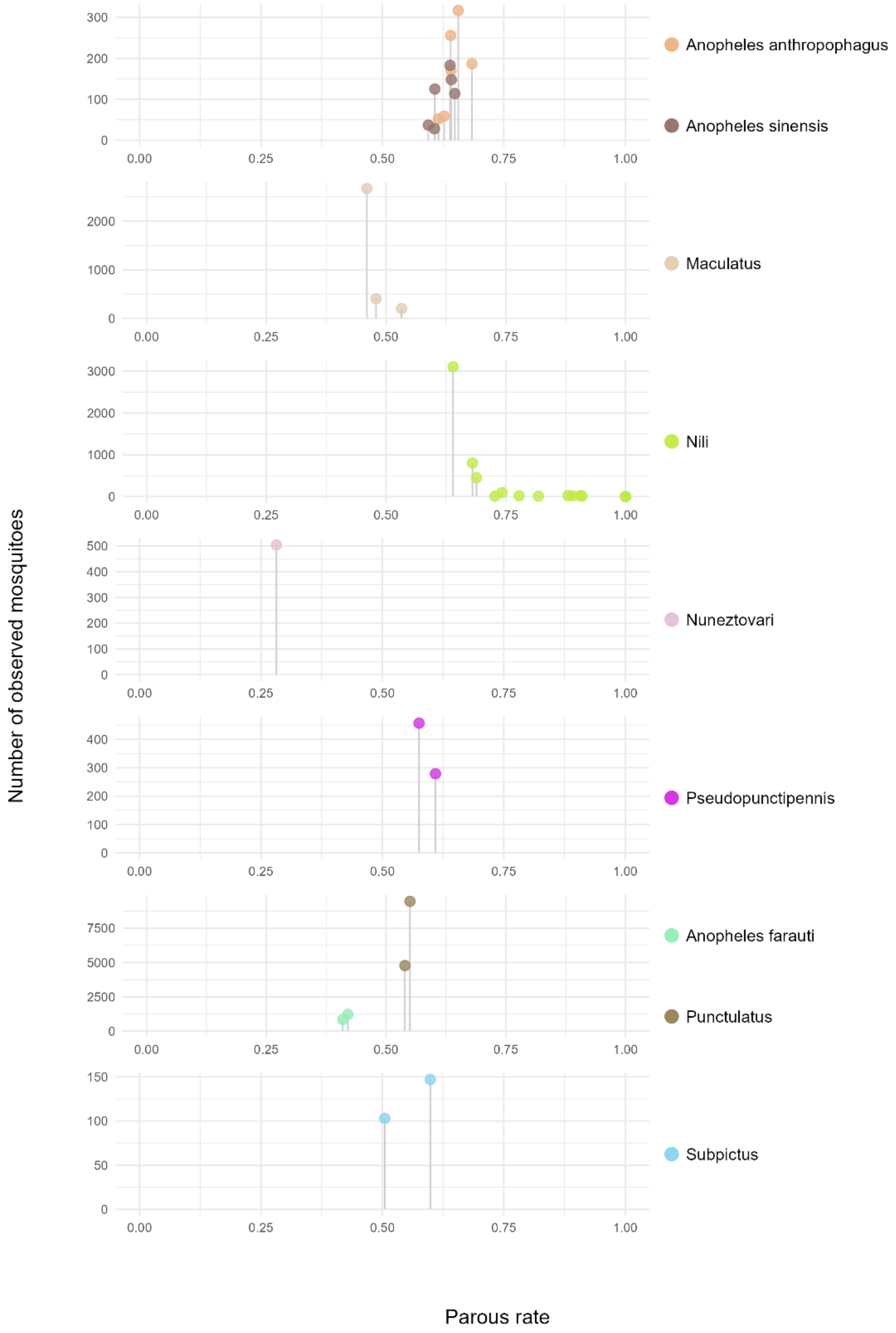

Supplementary Figure S11 (cont.). Available data by complexes and species for parous rate.

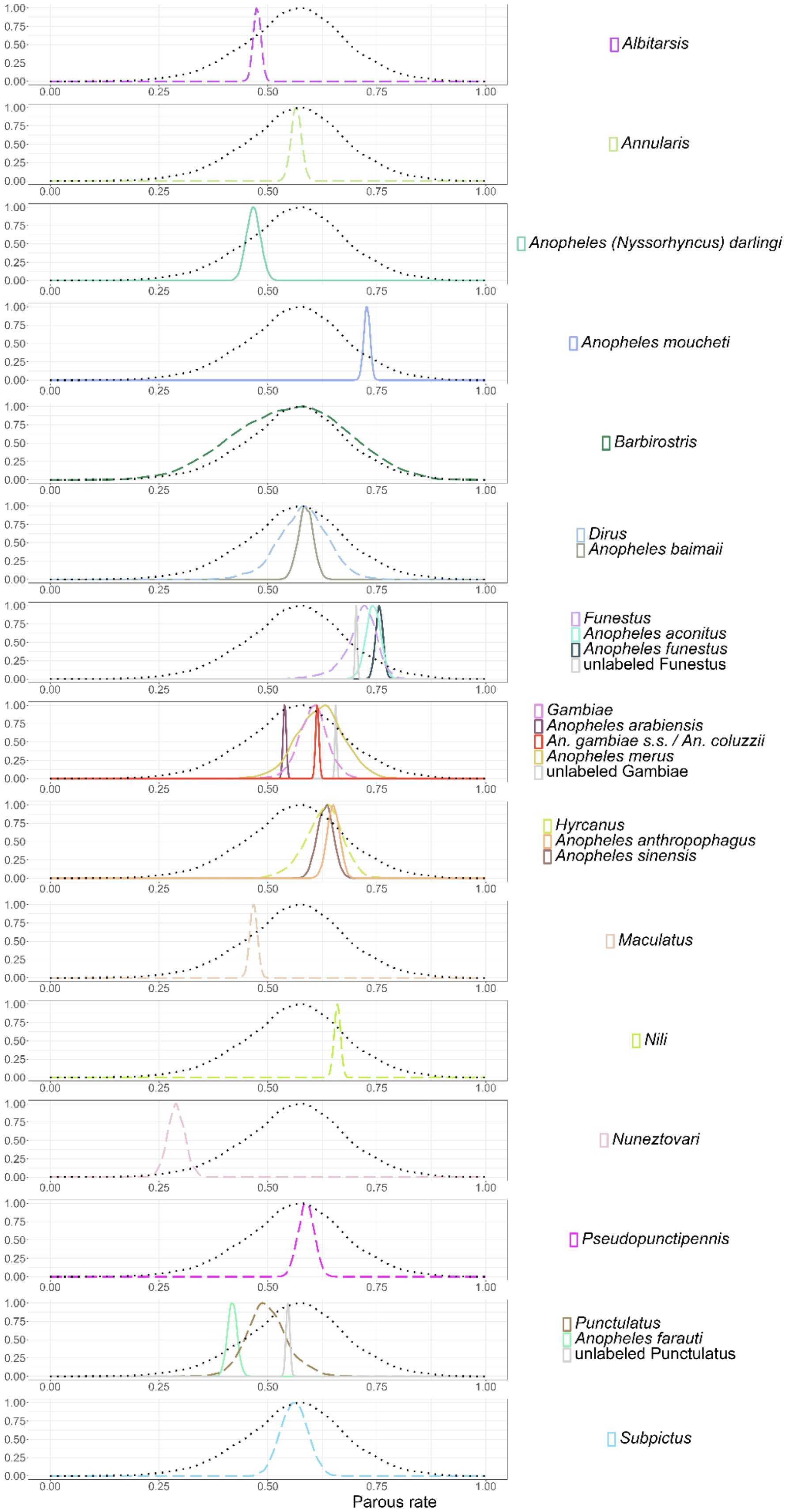

Supplementary Figure S12. Posterior densities for the parous rate. The black dotted line is the posterior density for the pooled estimate, the coloured dashed lines, for each complex, and the solid coloured lines for individual species. Y-axis is normalised to ease visualisation of all curves.

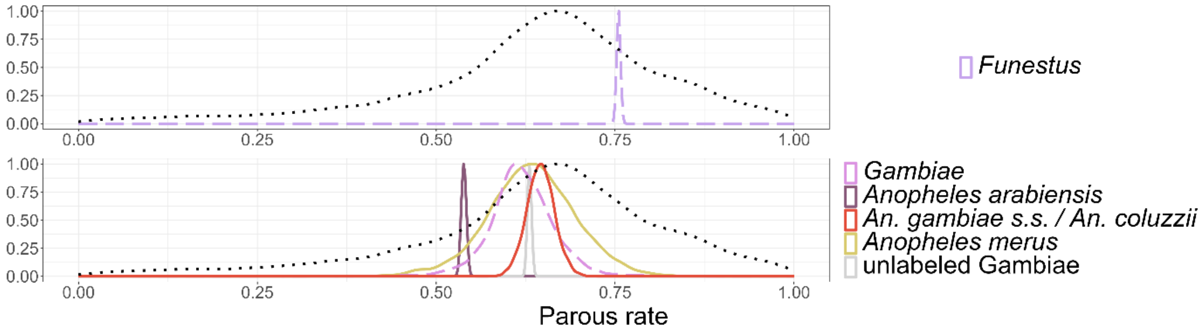

Supplementary Figure S13. Posterior densities for parous rate in East Africa. The black dotted line is the posterior density for the pooled estimate, the coloured dashed lines, for each complex, and the solid coloured lines for individual species. Y-axis is normalised to ease visualisation of all curves.

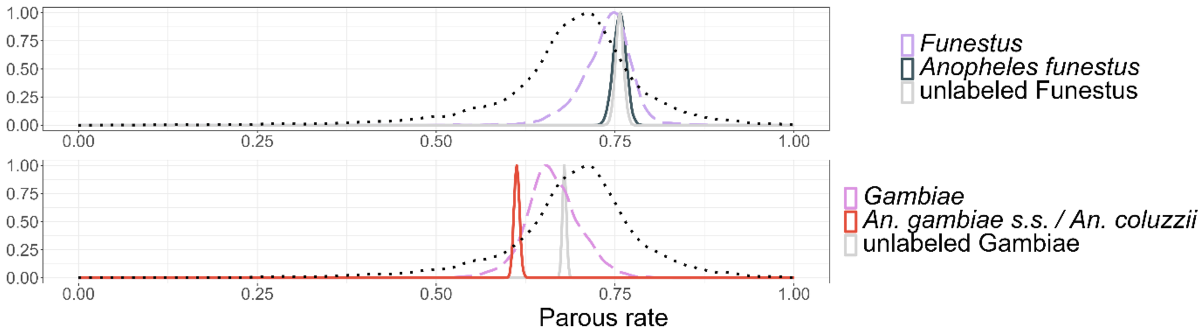

Supplementary Figure S14. Posterior densities for parous rate in West Africa. The black dotted line is the posterior density for the pooled estimate, the coloured dashed lines, for each complex, and the solid coloured lines for individual species. Y-axis is normalised to ease visualisation of all curves.

Resting Duration

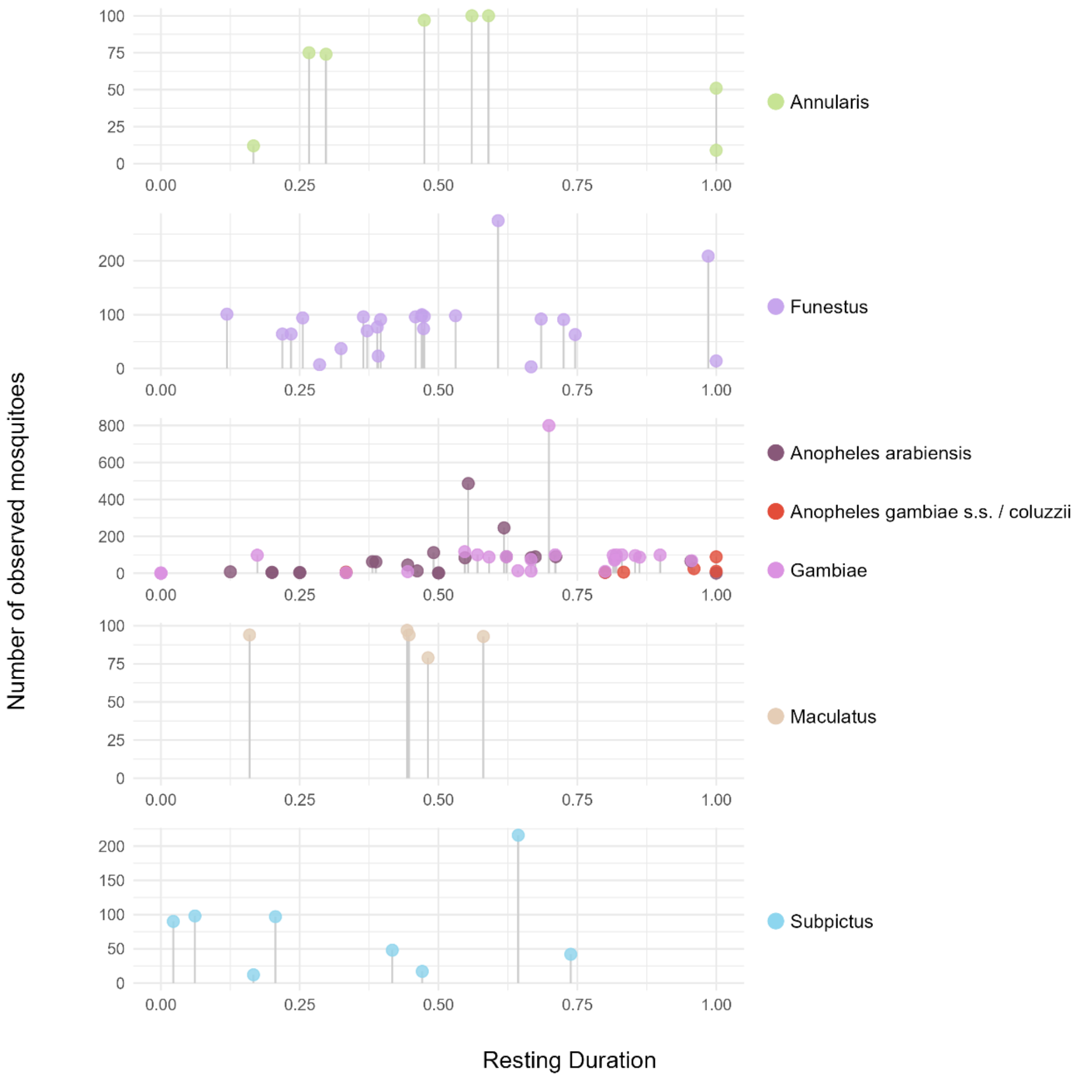

Supplementary Figure S15. Available data by complexes and species for resting duration.

Sac rate

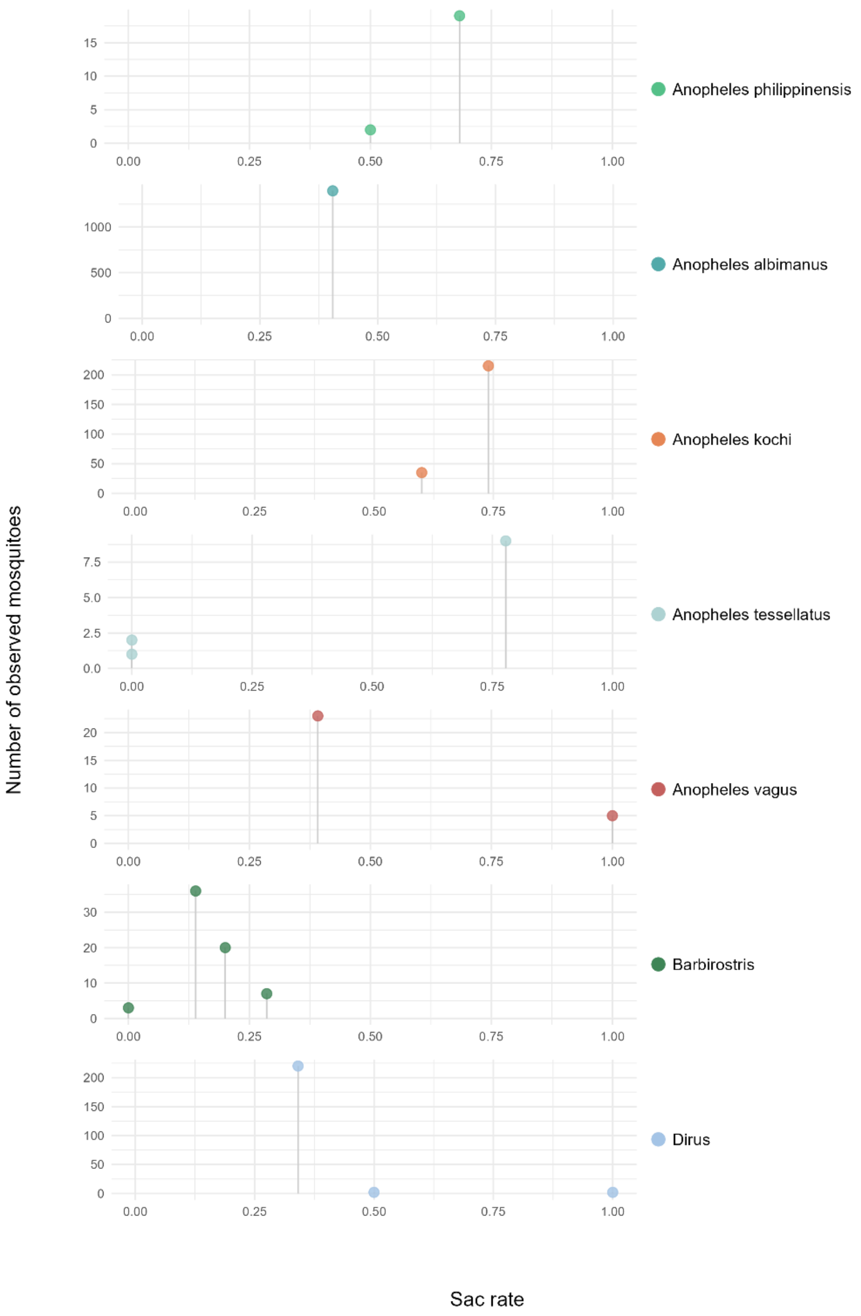

Supplementary Figure S16. Available data by complexes and species for the sac rate.

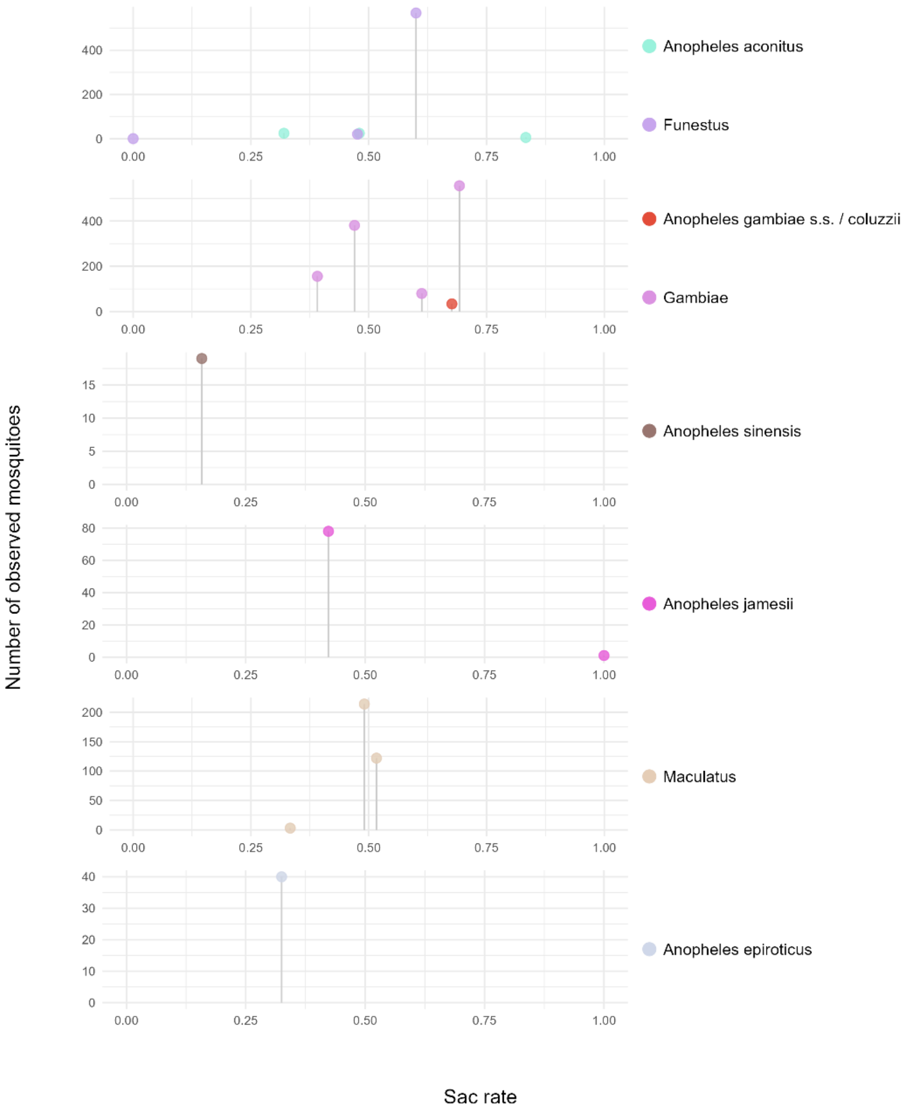

Supplementary Figure S16 (cont.). Available data by complexes and species for the sac rate.

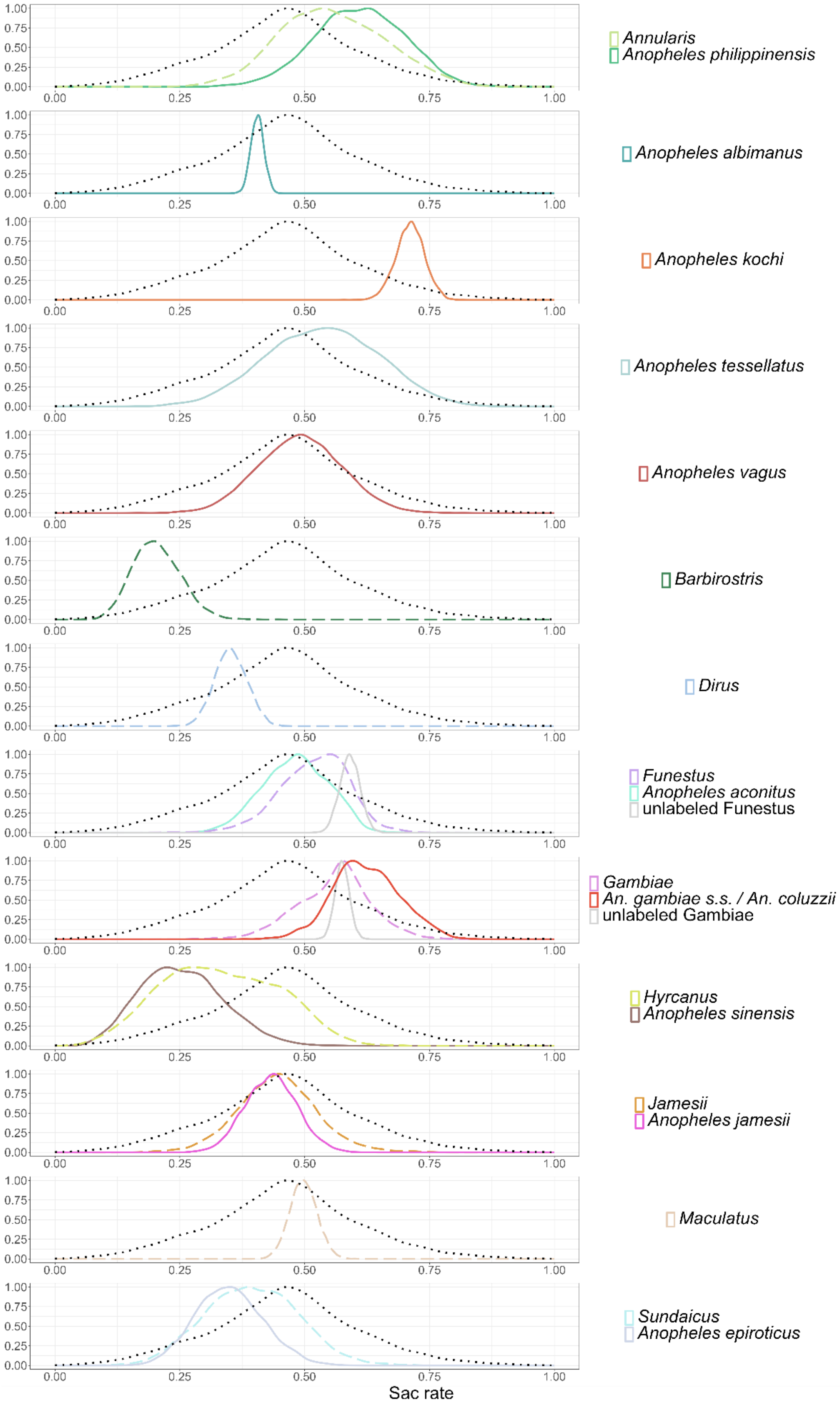

Supplementary Figure S17. Posterior densities for the sac rate. The black dotted line is the posterior density for the pooled estimate, the coloured dashed lines, for each complex, and the solid coloured lines for individual species. Y-axis is normalised to ease visualisation of all curves

3. Comparison of Bayesian estimates with empirical means

In this section, we compare the values of the Bayesian estimates with the raw empirical mean (weighted by sample size) of the available data.

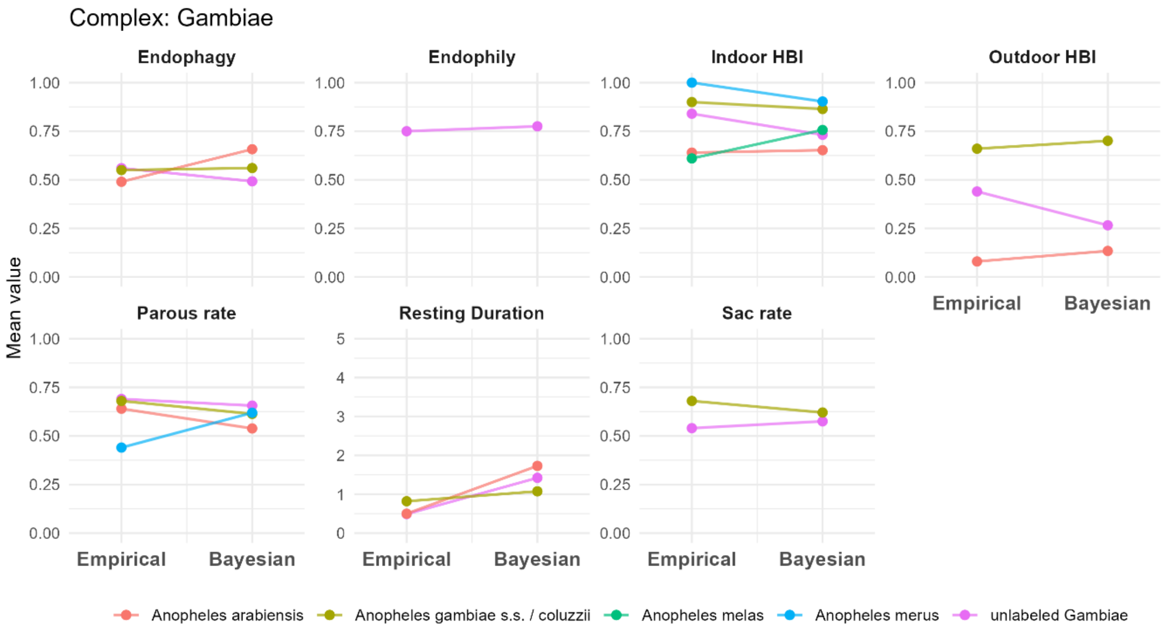

Supplementary Figure S18. Comparison of empirical and Bayesian estimates for the bionomics of key species in the Gambiae complex.

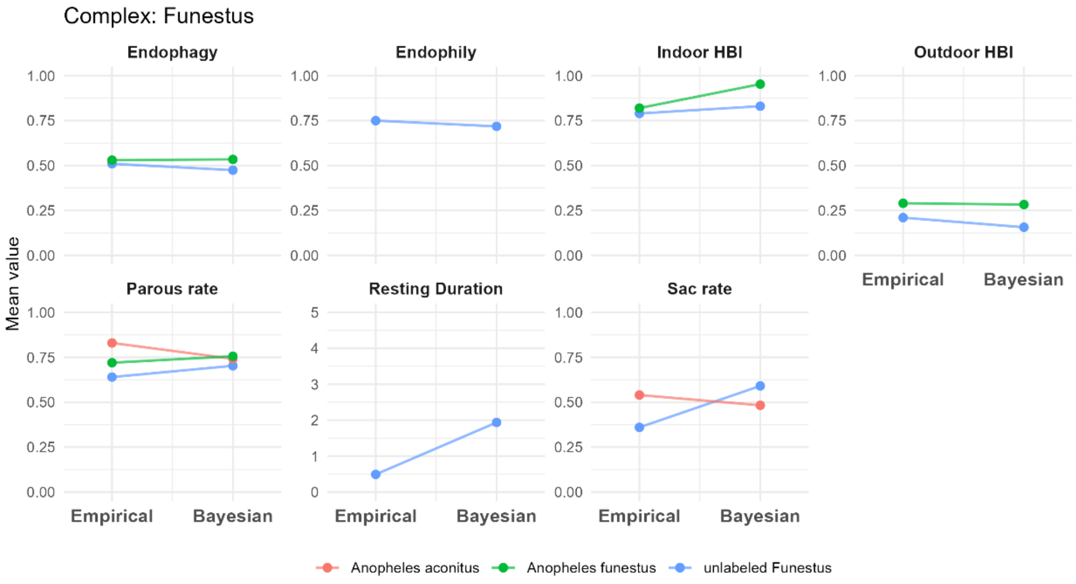

Supplementary Figure S19. Comparison of empirical and Bayesian estimates for the bionomics of species in the Funestus complex.

4. Vectorial Capacity reduction

Parameter	Description	Value
Reduction in host availability	From (Odufuwa in prep), as fitted in (Champagne et al. 2025)<br><br>Linear decay between unwashed and washed values over 3 years.	0.44 (unwashed) and 0.34 (washed)
Pre-prandial killing effect	From (Odufuwa in prep), as fitted in (Champagne et al. 2025)<br><br>Linear decay between unwashed and washed values over 3 years.	0.44 (unwashed) and 0.34 (washed)
Post-prandial killing effect	From (Odufuwa in prep), as fitted in (Champagne et al. 2025)<br><br>Linear decay between unwashed and washed values over 3 years.	0.17 (unwashed) and 0.35 (washed)
Attrition	Weibull distribution fitted with Martin et al. (2024)(Martin et al. 2024) data.	Half-life = 2.23<br><br>Shape parameter = 1.34
Coverage/Usage	Proportion of individuals sleeping under an ITN.	80%
Exposure	Using data and method form Golumbeanu (Golumbeanu et al. 2024)	Average of all species is 0.715%

Supplementary Table S2 Parameters used to calculate the reduction in vectorial capacity.

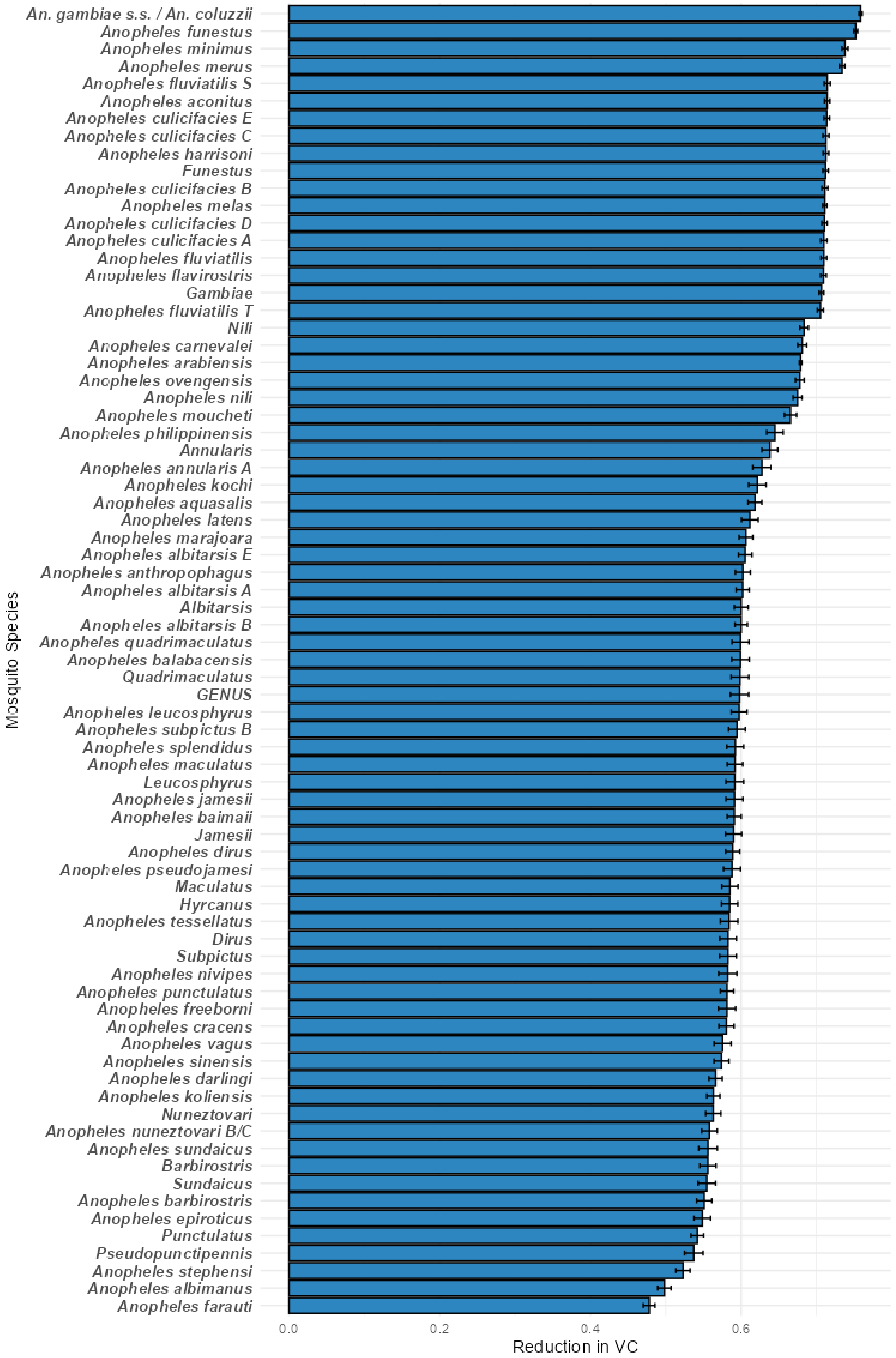

Supplementary Figure S20. Average reduction in vectorial capacity following an 80% coverage with chlorfenapyr-alphacypermethrin ITNs for all the mosquito species and complex with corresponding 95% confidence intervals.

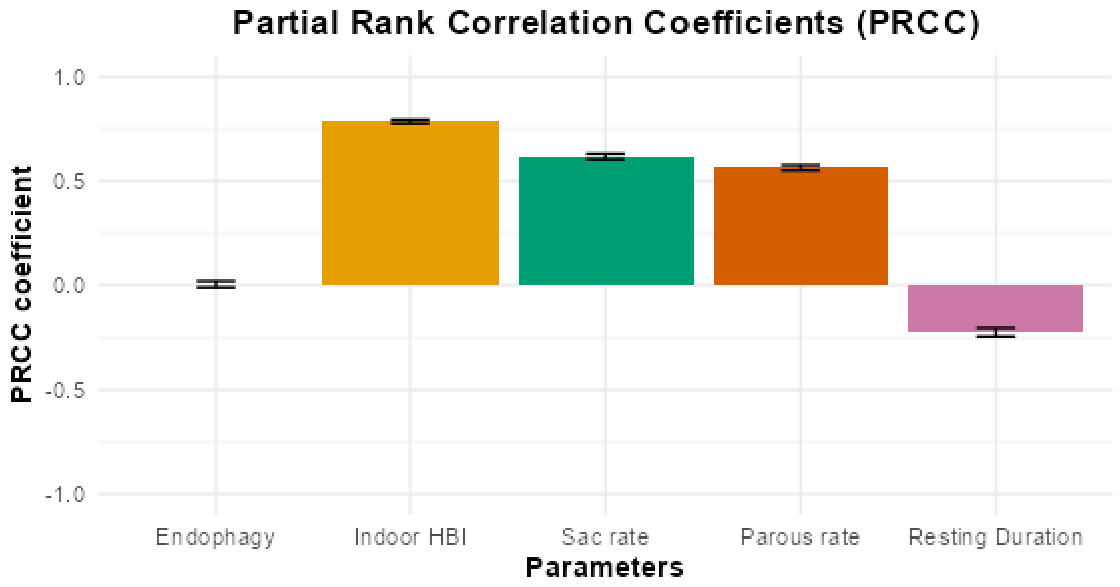

Supplementary Figure S21. Partial Rank Correlation Coefficients (PRCC) on the reduction in vectorial capacity after deployment of chlorfenapyr-alphacypermethrin ITNs with 80% coverage.

5. References

Champagne, Clara, Jeanne Lemant, Alphonce Assenga, et al. 2025. « Cascades of Effectiveness of New-Generation Insecticide-Treated Nets against Malaria, from Entomological Trials to Real-Life Conditions ». Prépublication, medRxiv, février 8. https://doi.org/10.1101/2025.02.07.25321565.

Golumbeanu, Monica, Olivier Briët, Clara Champagne, et al. 2024. « AnophelesModel: An R Package to Interface Mosquito Bionomics, Human Exposure and Intervention Effects with Models of Malaria Intervention Impact ». *PLOS Computational Biology* 20 (9): e1011609. https://doi.org/10.1371/journal.pcbi.1011609.

Martin, Jackline, Eliud Lukole, Louisa A. Messenger, et al. 2024. « Monitoring of Fabric Integrity and Attrition Rate of Dual-Active Ingredient Long-Lasting Insecticidal Nets in Tanzania: A Prospective Cohort Study Nested in a Cluster Randomized Controlled Trial ». *Insects* 15 (2): 108. https://doi.org/10.3390/insects15020108.

Odufuwa, Olukayode G. in prep. « Yorkool® G5 – chlorfenapyr insecticide-treated nets demonstrated comparative non-inferiority efficacy to the first-in-class Interceptor® G2 in the experimental hut in Tanzania against wild Anopheles arabiensis mosquitoes ». Prépublication.
